# Differential Access to Park Space Based on Country of Origin within Miami’s Hispanic/Latino Population: A Novel Analysis of Park Equity

**DOI:** 10.3390/ijerph18168364

**Published:** 2021-08-07

**Authors:** Marco Lorenzo Allain, Timothy W. Collins

**Affiliations:** Department of Geography, University of Utah, 260 Central Campus Dr., Rm. 4625, Salt Lake City, UT 84112, USA; u1121076@umail.utah.edu

**Keywords:** environmental justice, urban parks, greenspace, race/ethnicity, Hispanic/Latino, spatial analysis

## Abstract

Some U.S.-based park equity studies reveal that affluent and White neighborhoods have privileged access to greenspace. In such studies in the U.S. and elsewhere, park access indicators are typically examined in relation to measures of income, housing tenure, and broad race/ethnicity categories (e.g., Hispanic/Latino, Black, and White in the U.S.). The treatment of people as monolithic ethnic groups in park equity research is potentially misleading, particularly in global cities where ethnic populations are highly heterogeneous. In this study, we assess inequities in access to park space within the diverse Hispanic/Latino population of the Miami Metropolitan Statistical Area (MSA) based on national origin. We specified multivariable generalized estimating equations to examine social correlates of area-weighted park access at the census tract level. Our first model includes a variable for the proportion of the tract population of Hispanic/Latino ethnicity, which we substitute in the second model with variables representing the proportions of the tract population from the most populous country-of-origin groups in the MSA applicable to the Hispanic/Latino population. Our first model indicates robust negative relationships for the proportion Hispanic/Latino and Black/African American variables with area-weighted park access, adjusting for median household income, renter-occupancy, and old and young population composition. Our second model indicates negative relationships based on Cuban and Venezuelan neighborhood composition, while the four other country-of-origin subgroup variables exhibit statistically non-significant associations with area-weighted park access. Study findings have implications for the analysis of ethnicity categories in park equity research and interventions to promote park equity, especially in global cities.

## 1. Introduction

Urban public parks offer tangible and intangible benefits for nearby communities. Public parks act as green urban infrastructure that promotes public health through improved mental and physical well-being and by mitigating urban heat and flood hazards; public parks link transportation networks, enhance property values, host community social activities, and foster a sense of belonging and place for residents [1]. Among the various types of urban open spaces, public parks operated by local governments are distinctive because they are freely available to the public for active and passive recreation. For communities to benefit from public parks, they must be accessible. Accessible greenspaces are safe, well-maintained, and designed to accommodate the local community’s needs [2].

While some environmental justice (EJ) research shows that low socioeconomic status (SES) and minority communities have less access to greenspace in the U.S., other studies have found that urban, lower-income and racial/ethnic minority populations live closer to public parks [3,4,5,6]. Research is increasingly shedding light on park and greenspace inequities, yet there is currently limited knowledge regarding racial/ethnic disparities in access to local park space within the U.S. Additionally, it is important to recognize that the decreased size and quality of parks may serve to diminish the benefits of greater proximity for socially disadvantaged communities in some urban contexts [5]. Therefore, we need novel research that addresses questions of park equity based on a more nuanced examination of residents’ racial/ethnic status while accounting for park size or quality.

In this study, we advance upon previous park equity by examining neighborhood-level inequities in access to park space within the diverse Hispanic/Latino population of the Miami, Florida Metropolitan Statistical Area (MSA). We premise our analysis upon the recognition that the Miami MSA’s Hispanic/Latino population is highly heterogeneous—especially based on national origin and stratified experiences of immigration—making any assumption of homogeneity in park access based on this ethnic category problematic. Our focus on within-ethnic group differences in park access based on another axis of inequality (i.e., national origin) has implications for the treatment ethnicity in park equity research more broadly, in the U.S. and elsewhere. Additionally, in examining inequities in park access within the Miami MSA’s large and diverse Hispanic/Latino population, we introduce a novel measure of park access which weights access based on each park’s areal coverage and proximity to each neighborhood. As an advance upon pure proximity-based indicators of park access that prevail in the literature [7], our area-weighted park access measure enables us to assess inequities in access to park space.

### 1.1. Intracategorical Environmental Justice Analysis

A fundamental premise of our analysis is that the racial/ethnic classification used in all prior studies of park equity masks a great deal of variability, such that it may obscure to a greater degree than reveal ethnicity-based inequities. The *Revisions to the Standards for the Classification of Federal Data on Race and Ethnicity* established the current U.S. policy for collecting and presenting data on race/ethnicity for all official reporting purposes [8]. The U.S. Census Bureau-administered American Community Survey, for example, gauges ethnic and racial categories through two separate questions (“Are you Hispanic or Latino?” and “What is your race?”). Thus, respondents can identify as being Hispanic/Latino or not. They can also identify as being of five pre-defined racial categories (“American Indian or Alaska Native”, “Asian”, “Black or African American”, “Native Hawaiian or Pacific Islander”, and “White”) and of “Another race” (open-ended). Those federally defined categories have primarily determined the analysis and interpretation of racial/ethnic differences in U.S.-based EJ studies and the social sciences more broadly [9,10].

Intersectionality scholars have taken the lead on examining variability in experiences *within* those officially delineated racial/ethnic categories and clarifying how other axes of oppression interact with ethnicity to shape inequalities. McCall [11] laid out what she termed an *intracategorical* approach as a component of her work on intersectionality, which focused more broadly on the life experiences of multiply marginalized groups. Following McCall [11], an intracategorical analysis approach focuses specifically on characterizing differences of experience for subgroups within a category based on other axes of marginality. Here, for example, we apply an intracategorical approach to analyze differences in park area access based on national origin within the Hispanic/Latino category across Miami MSA census tracts. National origin reflects stratified experiences of immigration and is thus an important dimension of oppression in the Miami MSA (and other global cities with diverse immigrant populations) that intersects with Hispanic/Latino status to structure inequalities. Our intracategorical analysis approach offers an important contribution to park equity studies as it is applicable to examining intersectional factors shaping park access in contexts beyond the Miami MSA.

An intracategorical analysis approach was first applied in quantitative EJ research by Collins et al. [12], who comparatively examined inequities in exposure to air pollution across small areas in El Paso, Texas based on social axes difference within the Hispanic/Latino and non-Hispanic White populations. Subsequent intracategorical EJ analyses have documented inequities in exposure to air pollution based on key axes of difference within U.S. Asian, American Indian/Alaska Native, Black/African American, and White populations [13,14,15].

As a study site for intracategorical EJ analyses, the Miami MSA is particularly relevant owing to its high levels of racial residential segregation, as well as its highly diverse Hispanic/Latino population, which includes large groups of people from countries of the Caribbean as well as Central and South America. The Hispanic/Latino population of the Miami MSA is characterized by ranging levels of wealth, educational attainment, language proficiency, and exposure to hazards. Two intracategorical EJ studies, both of which examined air pollution, have focused on the Miami MSA. Grineski et al. [16] and Chakraborty et al. [17] both found that Cuban status (vs. non-Hispanic White status) was associated with greater exposure to traffic-related air pollution, while other country-of-origin statuses were not. This was partly due to the Cuban population concentration in downtown Miami.

Hispanic/Latino status is not uniformly associated with social marginality in the Miami MSA, as it is in some other parts of the U.S. In Miami, Hispanic/Latino status intersects in important ways with national origin to shape experiences of inequality. Mexicans are historically a highly marginalized group, and they are residentially concentrated nearer to agricultural lands on the metropolitan fringe [16]. Cubans experience relative economic and political privilege in the MSA due to their unique migration history. The Cuban Adjustment Act of 1966 made acquiring a visa and becoming a naturalized U.S. citizen considerably more accessible and faster for Cuban immigrants than other immigrant groups. Cubans, Venezuelans, and Colombians have increasingly concentrated in urban core neighborhoods of the MSA. Colombian migrants have tended to settle within distinct concentrations near Cuban neighborhoods. Many migrants from Venezuela have arrived after fleeing the country’s economic and political crisis. A relatively high proportion of Venezuelan migrants lack legal U.S. residency status, and many have settled in the suburban community of Doral located northwest of Miami proper. People of Puerto Rican descent have a long history in the MSA and exhibit a more dispersed pattern of settlement. Thus, based on the differentiated residential trajectories of these groups, it is likely that Hispanic/Latino status has intersected with national origin to shape inequities in access to park space in the Miami MSA. However, no prior studies have analyzed intersectional park inequities in Miami or elsewhere.

In sum, U.S.-based quantitative EJ research has relied almost entirely on examining the broad, federally-defined racial-ethnic classification categories, such that the recent wave of intracategorical EJ research on air pollution has expanded our understanding of racial/ethnic environmental inequities masked in prior analyses. However, no previous research has applied an intracategorical approach to examining park or greenspace access inequities, representing a substantial gap in knowledge. In this study, we address that gap by examining small area inequities in park access across the Miami MSA based on Hispanic/Latino ethnic status and intracategorical inequities in park access within the Hispanic/Latino population based on country-of-origin, which is the predominant mode of reporting identity among Hispanic/Latino people in the U.S. [9].

### 1.2. Park Access and Equity

Park accessibility is an established topic within leisure/recreation studies and geography [18]. Location theory, which derives from economic geography and regional studies, provides a basis for traditional park accessibility studies. Applied to park accessibility, the location theoretic approach has traditionally focused on optimizing proximity to parks for potential users while minimizing operational costs [6,19]. Thus, urban park studies have tended to emphasize physical and economic variables that influence park accessibility and utilization in spatial terms [19]. This traditional approach to park accessibility research does not account for multidimensional social geographic factors that shape park access and equity in cities.

In terms of the social factors influencing park access, studies in the U.S. have mainly examined the broad racial/ethnic categories, socioeconomic status indicators, and demographic variables available from the U.S. Census-administered American Community Survey. Based on simple measures of proximity, several studies have found, counterintuitively, that Black/African American or Hispanic/Latino groups lived closer to parks than White populations [3,4,6,20,21]. In contrast, Rigolon and Flohr [22] found that the White group lived closer to parks in Denver, Colorado. Other studies have found that the ease of access and size of the park were especially important influences on racial/ethnic minority residents’ preferences for and use of municipal parks [18,23,24,25]. Findings from studies of the influence of socioeconomic status on proximity to parks have been mixed [20,22], although several studies have found lower income and renter-occupancy (vs. owner-occupancy) to be associated with less overall greenspace access [23,25,26,27,28,29]. In studies focused on age-based park inequities, Cutts et al. [3] found that children (less than 18 years of age) had significantly less park access in Phoenix, Arizona, while Nicholls [4] found that children had greater park access in Bryan, Texas. In terms of inequities based on older age status, Guo et al. [27] found that residents 60 years and older in Beijing, China had less park access than those below the age of 60.

Geospatial assessment of access to parks is an ongoing challenge in park equity studies. Most studies of park equity assume that accessible parks are within a walkable distance of one’s home because visitation to parks beyond a walkable distance demands another mode of transit [1]. If a park is not accessible by foot, users typically view it as a destination, which reduces their likelihood of using it for unplanned exercise or leisure. Accessibility is relative, however, such that walkability for children and the elderly may depend on parks being closer.

In the park access literature, spatial accessibility has been gauged using a range of measures that incorporate, for example, Euclidean distance to parks, network distance to parks, park area per capita, or the number of parks per area [3,4,30,31]. Additionally, measures of park quality have received increasing attention in park equity research. Studies examining park size and quality have found more consistent race/ethnicity- and socioeconomic status-based inequities than those focused on park proximity alone. Higher quality parks—for example, those that are larger or with greater tree canopy cover—tend to be more accessible to socially privileged communities both within and beyond the U.S. [32,33,34]. In the U.S., for example, a bevy of studies have found significantly more park area accessible to higher (vs. lower) SES and White (vs. Black, Hispanic/Latino, and Asian or Pacific Islander) groups [35,36,37,38].

### 1.3. Research Questions

In this study, we advance knowledge by examining inequities in park access based on race/ethnicity, SES, and age in the Miami, Florida (USA) Metropolitan Statistical Area (MSA) using an area-weighted park access metric at the neighborhood (i.e., census tract) level. This is the first park equity study to employ an intracategorical EJ approach focused on inequities within the diverse Hispanic/Latino population. We address the following research questions:

1. Are there neighborhood inequities in area-weighted park access based on race/ethnicity, socioeconomic status (SES), and age in the Miami MSA?
2. Are there neighborhood inequities in area-weighted park access within the Hispanic/Latino population based on country-of-origin in the Miami MSA?

Based on the EJ and park equity literature findings, we hypothesize that higher proportions of non-White racial/ethnic residents, lower SES, and higher proportions of children and older adults in census tracts are associated with less area-weighted park access. Based on the intracategorical EJ literature on air pollution [12,16,17], we hypothesize that there are within-group differences in park access based on country-of-origin for the Miami MSA’s Hispanic/Latino population.

## 2. Materials and Methods

### 2.1. Dependent Variable: Area-Weighted Park Access

We identified city and county public parks using the USA Parks GIS layer produced by Esri Data and Maps, as well as from parks layers obtained from local governmental agencies in the Miami MSA [39,40,41,42,43,44]. All national and state parks in the Miami MSA were removed from the analysis such that only city and county parks were included. National and state parks are located on the periphery of the Miami MSA, are accessible almost exclusively by automobile, have use fees, and are subject to different accessibility conditions than the county and municipal parks. For example, while users of municipal parks are primarily local residents, out-of-state and international tourists account for approximately 75% of individual visits to Everglades National Park [45]. Additionally, we omitted public (and private) golf courses from the study because they implement use fees and have accessibility conditions distinct from those of municipal and county parks. We removed duplicate records of parks present across multiple data sources.

We utilized spatial analytic techniques to develop a measure of census tract-level access to park area that addresses limitations of park access measures used in prior studies. Most studies of park equity have used either proximity-based indicators or spatial coincidence methods to measure park access [7]. As noted above, pure proximity-based indicators neglect park area or quality. As examples of spatial coincidence methods, Abercrombie et al. [46] measured park access by calculating the number of parks with boundaries intersecting each census tract, while Vaughn et al. [47] assessed access to park area by summing the area of all parks intersecting each tract. The spatial coincidence method is problematic and considered outmoded in environmental justice research for several reasons. First, it does not account for boundary or edge effects. For example, a given park may be close to but not intersect with the boundary of a tract, such that the access of the park to that tract is unmeasured. This is particularly problematic in dense urban areas such as the Miami MSA wherein units such as census tracts are small in area. Second, this method assumes that access to a park is uniform within intersecting tracts and restricted only to intersecting tract boundaries. Predefined geographic entities or census units, however, do not represent the actual access for an area or population. More advanced methods, such as those of Bolin et al. [48], Grineski et al. [49], and Flores et al. [50], account for boundary effects and non-uniform access through the calculation of indices using distance-based buffers around parks and areal apportionment to census tract boundaries. 

We developed our approach to measuring area-weighted park access based on those more advanced areal apportionment methods. First, we buffered each park feature to assess park proximity. Buffering techniques have been used to characterize proximity in many EJ studies [51]. We created a buffer of 1.5 km or 0.93 miles around each park feature, which roughly equates to a 15 min walk on foot with urban traffic [52]. This distance-based criterion for park access has been used in prior park equity research (e.g., [53]).

**Figure 1:**
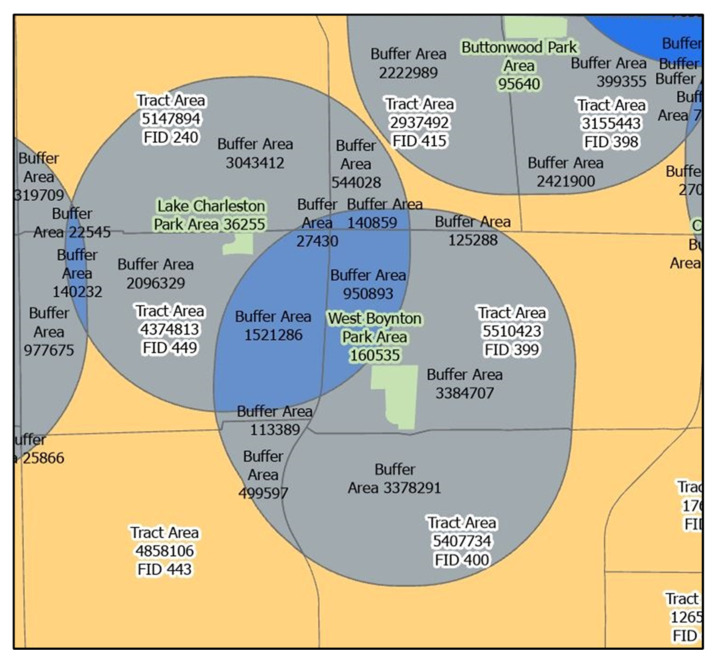
Map illustrating spatial elements of the area-weighted park access calculation, including park boundaries and areas, tract boundaries and areas, and intersecting park buffers and areas. Note: FID elements (e.g., “FID 400”) are unique identifiers for each census tract.

The U.S. Census Bureau produces demographic data at the census tract level that has been used in many park equity studies [6,7,30,54]. We intersected the buffered parks layer with the census tract boundary layer using the “Intersect” Analysis tool in ArcPro version 2.6 (Esri, West Redlands, California, USA). We then divided the area (in meters squared) of each piece of park buffer intersecting each tract by the area of the tract (in meters squared) to estimate the proportion of the tract with access to each park. Next, to weight access to each park in each tract by the total areal coverage of the park, we multiplied the proportion of the tract with access to each park (which we calculated in the previous step) by the respective area of each park in meters squared. We then summed the area-weighted park access value for every park in each tract using the “Summarize Within” Analysis tool in ArcPro version 2.6 (Esri, West Redlands, California, USA). This created the tract-level area-weighted park access dependent variable we used in the analysis. The unit of the dependent variable is in estimated square meters of the accessible park area. This formula specifies the calculation:

(1) $$Area\;Weighted\;Park\;Access=\overset{n}{\underset{Ba}{\sum }}\frac{Ba}{Ta}Pa\;$$

where *Ba* is the buffer area intersecting the tract, *Ta* is the tract area, *Pa* is the park area, and *n* is the sum of additional terms for each park buffer intersecting a given tract. Figure 1 illustrates the area-weighted park access calculation. For example, we calculated area-weighted park access for census tract FID 399 in Figure 1 as: (3,384,707/5,510,423 × 160,535 (West Boynton Park Area)) + (950,893/5,510,423 × 36,254 (Lake Charleston Park Area)) = 104,863 estimated square meters of accessible park area.

### 2.2. Independent Variables: Social Indicators

We used 2014–2018 American Community Survey (ACS) five-year estimates to create variables for race/ethnicity, country-of-origin groups, socioeconomic status (SES), and age at the census tract level. We used variables previously examined in EJ studies and additional national origin variables, which have been examined in only a few EJ studies focused on air pollution (e.g., [19]). There are a total of 1192 census tracts included in the study. We excluded 27 census tracts across the three counties that comprise the study area because they had fewer than 500 residents or 200 occupied housing units.

In terms of race/ethnicity, we included variables representing the proportions of the tract population that were Hispanic/Latino, non-Hispanic Black, non-Hispanic Asian, non-Hispanic Native American, non-Hispanic Pacific Islander, and non-Hispanic of another race or multiple races (other/multi race). The proportion of the population that was non-Hispanic White serves as the reference for those variables. To examine within-Hispanic/Latino distinctions in relationships with accessible park space based on national origin, we included variables for the proportions of the tract population that were of Mexican, Puerto Rican, Cuban, Colombian, and Venezuelan origin to represent the five most populous national origin groups in the MSA for the Hispanic/Latino population. We also included a sixth variable that represents the proportion of the population from all other countries of origin for the Hispanic/Latino population. The proportion of the population that was non-Hispanic White is the reference for the national origin variables.

Additionally, we examined two measures of tract-level SES, including median household income and the proportion of renter-occupied housing units. We also included two variables to assess the effects of youth and older age composition on area-weighted park access. Specifically, we examined measures of the proportions of the census tract population under 18 and over 64 years of age, respectively, in reference to the proportion of the population aged from 19 to 64 years of age.

### 2.3. Analysis Approach 

First, we conducted a univariate analysis by calculating descriptive statistics and mapping the area-weighted park access variable and key independent variables. We then conducted multivariable analyses using generalized estimating equations (GEEs) with robust covariance estimates. We selected GEEs because, first, they accommodate clustered and non-normally distributed data [55]. The tract-level distribution of area-weighted park access exhibits a non-normal distribution and distributions of several independent variables do not approach normality. Second, GEEs are preferable to other modeling approaches that account for non-independence of data (e.g., hierarchical linear models) because GEEs estimate unbiased regression coefficients when using a robust covariance estimator, even if the correlation structure is misspecified [55]. Third, because our focus is on neighborhood-level effects, not on higher-level effects, GEEs are appropriate because they adjust for clustering as a nuisance [56]. Prior EJ studies have used GEEs to address geographic clustering [10,13,15,57,58].

In our GEEs, we defined clusters of census tracts based on their county of location (i.e., Broward, Miami-Dade, or Palm Beach) by their median year of housing construction category (i.e., “2000 or later”, “1990–1999”, “1980–1989”, “1970–1979”, “1960–1969”, “1950–1959”, “1940–1949”, or “1939 or earlier”), which yielded 20 clusters. To address missing values for the year of housing construction variable in the 2018 ACS 5-year estimates for 49 tracts, we included values for those corresponding tracts from the 2017 and 2016 ACS 5-year estimates. Prior environmental equity studies have used this cluster definition [13,15,57,58].

We estimated two GEEs predicting area-weighted park access. Model 1 is the base model, which includes the proportion Hispanic/Latino variable, in addition to the five other proportion racial/ethnic minority group variables (Black, Native American, Asian, Pacific Islander, Other), the young and old age composition variables (proportions under 18 and over 64 years of age), and the socioeconomic status variables (median household income and proportion renter-occupancy). For the race/ethnicity variables, results are interpretable in reference to proportion White. The age variable results are interpretable in reference to the proportion of the population aged 19–64 years. Results for proportion renter-occupancy are interpretable in reference to the owner-occupancy rate. Model 2 includes the six national origin proportion variables (Colombian, Cuban, Mexican, Puerto Rican, Venezuelan, and Other Hispanic/Latino origin) in place of the proportion Hispanic/Latino variable used in Model 1. Again, results for those variables are interpretable in reference to proportion White. Otherwise, Model 2 includes the same independent variables as Model 1.

We started by estimating GEEs with varying specifications to determine the best fitting models. This involved alternating the:

- correlation matrix specification from independent to exchangeable to unstructured
- distribution from normal to inverse Gaussian to gamma to Tweedie
- link function from logarithmic (log) to identity (linear)

After fitting GEEs using all possible combinations of those model specifications, we selected the models with the lowest quasi-likelihood under the independence model criterion (QIC) goodness-of-fit values. For Model 1, the exchangeable correlation structure with a gamma distribution and a log link function was the best fitting specification. The exchangeable correlation structure, Tweedie (index parameter = 1.5) distribution, and log link function had the best fit for Model 2. We present results from those models.

Additionally, to clarify the effects of key independent variables on area-weighted park access exhibited in Model 1 and Model 2, we calculated estimated marginal (EM) means. EM means provide value estimates of the dependent variable based on specified values for independent variables of interest, adjusting for all other GEE model specifications. Specifically, we calculated EM means at every 10th percentile (from 0 to 100) for each independent variable in Model 1 and Model 2 (separately) that was associated with significantly reduced area-weighted park access.

Based on condition index criteria, our inferences from the GEEs were unaffected by multicollinearity. We used SPSS Statistics v25 (IBM, Armonk, NY, USA) to conduct the analyses.

## 3. Results

**Figure 2:**
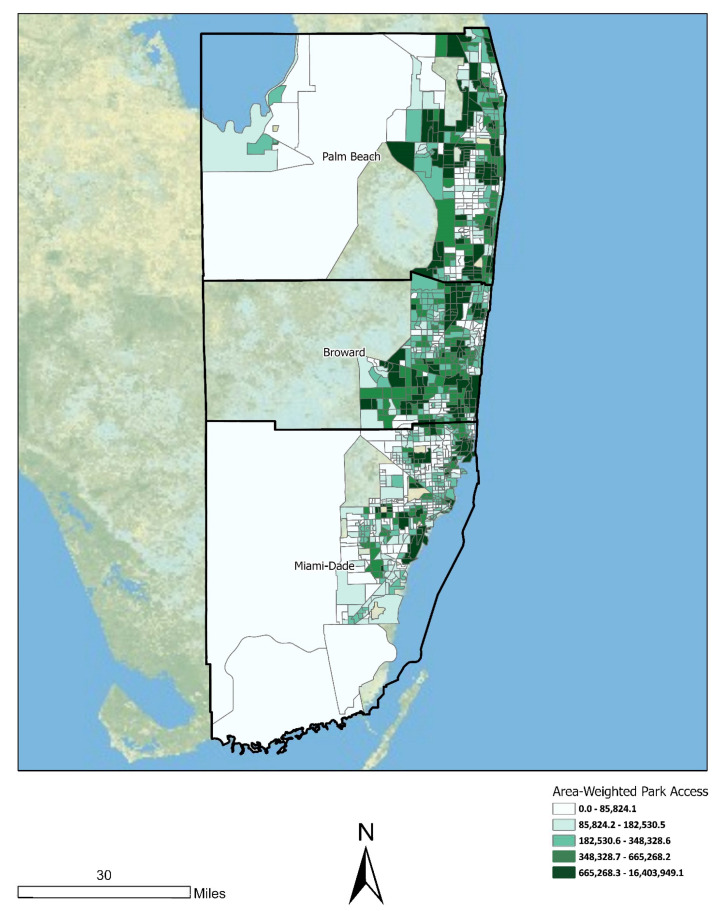
Spatial distribution of area-weighted park access across Miami MSA census tracts (*n* = 1192).

Figure 2 is a classified choropleth map that depicts the spatial distribution of the area-weighted park access dependent variable across the Miami MSA. For this map, we grouped census tracts into quintiles. Tracts in the highest quintile (top 20%) for area-weighted park access were located primarily in urbanized areas near the coast, particularly in Palm Beach and Broward Counties. Tracts in the lowest quintile were located primarily in rural areas in the western portion of the MSA as well as in Miami-Dade County.

**Figure 3:**
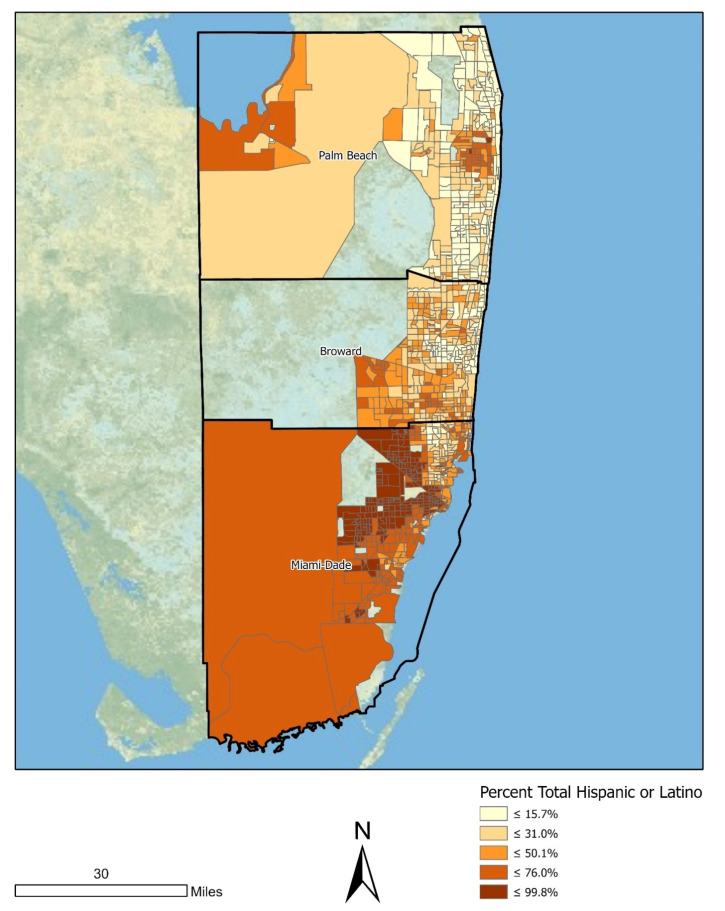
Spatial distribution of the percentage of residents of Hispanic/Latino ethnicity across Miami MSA census tracts (*n* = 1192).

The census tract distribution of the proportion Hispanic/Latino variable is shown in Figure 3, which is a quintile classified choropleth map. Tracts in the highest quintile for proportion Hispanic/Latino were concentrated in the entirety of Miami-Dade County and the Western portions of Palm Beach and Broward Counties. Tracts in the lowest quintile were located primarily in coastal Palm Beach County.

**Figure 4:**
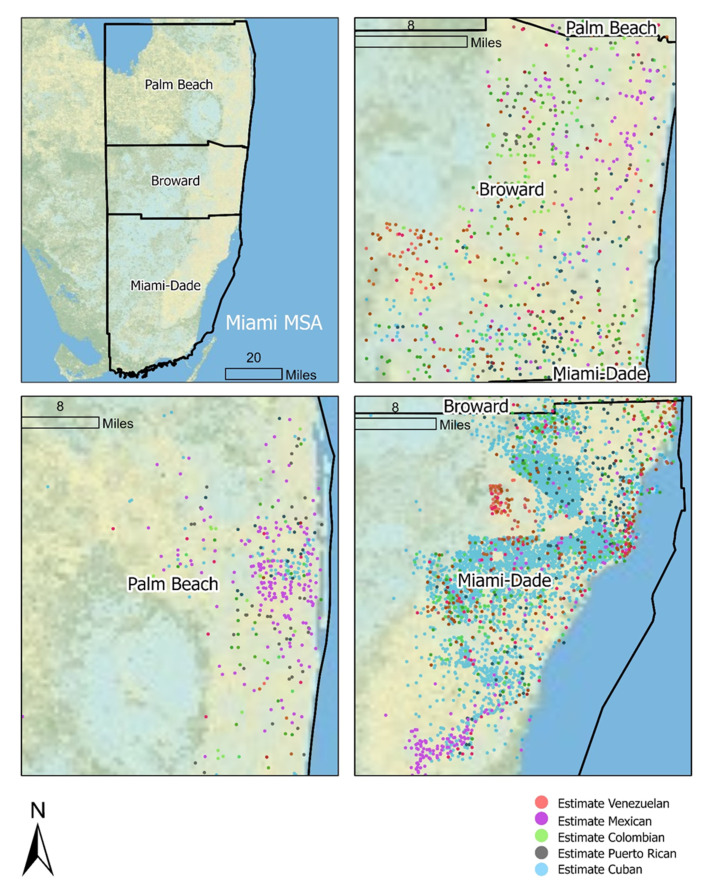
Spatial distribution of country-of-origin groups for the Hispanic/Latino population in the Miami MSA. Note: Each dot represents an estimated 200 residents from a group.

Figure 4 is a dot density map that shows the approximate (tract-level) residential locations of each of the national origin groups coded by color. Each dot represents the residential concentration of 200 or more members of a given national origin group. Cuban populations are located primarily in Miami-Dade County. Venezuelan populations are located near Cuban populations in Northern Miami-Dade and Central Broward County, and Mexican populations are located predominantly in rural Southern Miami-Dade.

Descriptive statistics for all analysis variables are included in Table 1. Area-weighted park access and median household income ranged substantially. The maximum values for proportion Hispanic/Latino, proportion Black, and proportion White were nearly one hundred percent, while the minimum values were near zero. Based on the mean values, Cuban exhibited the highest prevalence among country-of-origin groups, followed by Colombian, Puerto Rican, Mexican, and Venezuelan, respectively.

Table 2 summarizes our multivariable GEE results. In Model 1, significant and negative coefficients for the proportion Hispanic/Latino, non-Hispanic Black, and American Indian/Alaska Native variables indicated that tracts wherein these racial/ethnic minority groups were increasingly concentrated (relative to the proportion White) had less area-weighted park access.

**Figure 5:**
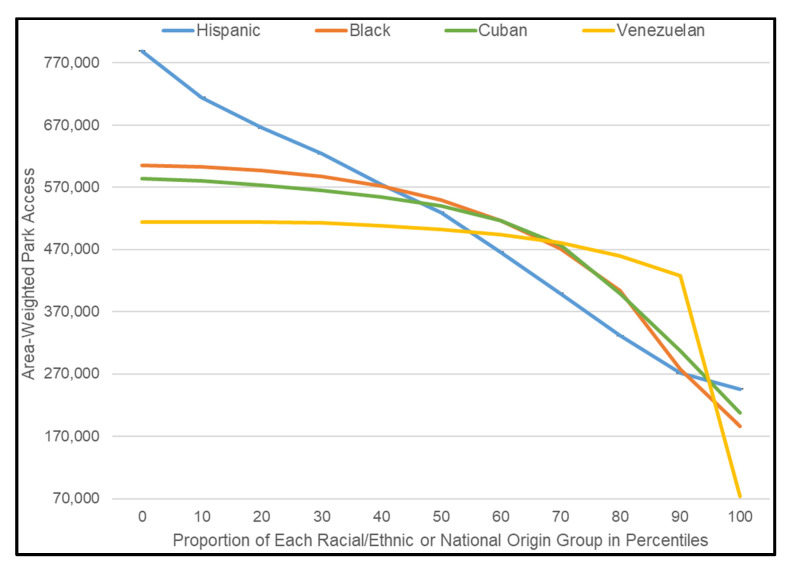
Estimated marginal mean area-weighted park access for key racial/ethnic and national origin variables (Table 2) when all other variables are at the mean.

EM mean results help describe the strength of those associations. Figure 5 graphs EM means for each independent variable in Model 1 and Model 2 that was associated with significantly reduced area-weighted park access. Holding all other independent variables in Model 1 constant, when proportion Hispanic/Latino was at the minimum (0), 50th percentile (0.339), and 100th percentile (0.994) for census tracts in the study area, the respective point estimates for area-weighted access were 788,726, 529,787, and 245,391 square meters. Thus, when the proportion of the tract population that was Hispanic/Latino was at the minimum vs. maximum compared to the proportion that was White, the estimated accessible park area was more than three times greater. Similarly, when the proportion Black was 0 (the case for 5.5% of metro Miami tracts) vs. at the 50th (0.079) and 100th (0.964) percentiles, the respective point estimates for area-weighted access were 604,667, 549,038, and 185,564 square meters. While the effect size of proportion American Indian/Alaskan Native was large, the vast majority of tracts (85.25) had zero values for the variable, such that EM mean results for this group were not illustrative and thus are not depicted in Figure 5.

Additionally, in Model 1, the proportion Other/Multi-Race variable showed a positive and significant association with area-weighted park access. However, “Other/Multi-Race” does not constitute a coherent racial/ethnic identity. Instead, it is a catch-all variable we included in the models to enable direct comparisons of associations for the other minority racial/ethnic group variables with the proportion of the tract population that was non-Hispanic White.

In Model 1, the significant and positive coefficient for the proportion of the tract population under 18 years of age indicated greater area-weighted park access relative to tracts with higher proportions of the population from 18 to 64 years of age (adjusting for the tract composition of residents older than 64 years of age).

In Model 2, we replaced the conventional proportion Hispanic/Latino variable with the six country-of-origin variables, which gauge the proportions of the population of Mexican, Puerto Rican, Cuban, Colombian, Venezuelan, and other Hispanic/Latino ancestry. Model 2 exhibited significant and negative coefficients for the proportion Cuban and Venezuelan variables, indicating that tracts wherein these national origin groups were increasingly concentrated had significantly less area-weighted park access relative to tracts where Whites were concentrated. The EM mean results depicted in Figure 5 clarify the strength of those associations. Holding all other independent variables in Model 2 constant, when proportion Cuban was at the minimum (0), 50th percentile (0.068), and 100th percentile (0.891) for census tracts in the study area, the respective point estimates for area-weighted access were 584,302, 539,693, and 207,247 square meters. Thus, when the proportion of the Cuban tract population was at the minimum vs. maximum compared to the proportion that was White, the estimated accessible park area was nearly three times greater. Similarly, when the proportion Venezuelan was 0 (the case for 31.7% of Miami MSA tracts) vs. at the 50th (0.007) and 100th (0.608) percentiles, the respective point estimates for area-weighted access were 514,208, 502,338, and 73,169 square meters. Results for the other Hispanic/Latino ancestry subgroups showed statistically non-significant associations with area-weighted park access. These results suggest that the neighborhood concentrations of metro Miami’s Cuban and Venezuelan populations drove the negative association between the tract-level proportion Hispanic/Latino variable and area-weighted park access exhibited in Model 1.

The other independent variables in Model 2 exhibited the same associations (in terms of direction and significance) as they did in Model 1 with area-weighted park access, with one exception. The proportion under 18 years of age variable, which exhibited a positive and significant association with area-weighted park access in Model 1, exhibited a positive and statistically non-significant association in Model 2.

## 4. Discussion

In reference to our first research question—Are there neighborhood inequities in area-weighted park access based on race/ethnicity, socioeconomic status (SES), and age in the Miami MSA?—we found that increasing tract proportions of Hispanic/Latino, Black, and American Indian/Alaska Native residents (in reference to the tract composition of White residents) were associated with significantly less area-weighted park access. Findings for those racial/ethnic variables generally align with some prior park equity studies. The proportion of the tract population under 18 years of age was associated with greater area-weighted park access relative to tracts with higher proportions of adults between 18 and 64. However, that effect was only statistically significant in one of the two GEE models. We found no significant associations between the SES variables (income and renter-occupancy) and area-weighted park access, which is not consistent with most prior studies of park equity. Thus, our results suggest that race/ethnicity more powerfully structures area-weighted park access than SES in the Miami MSA. One explanation for this pattern is the presence of many high-value coastal properties—with greater access to blue amenities (e.g., beach access)—in the Miami MSA. Montgomery et al. [59] found that higher neighborhood SES was associated with greater public beach access in the Miami MSA. Thus, for high SES neighborhoods along the coastline, improved access to blue space may provide greater total access to a recreational area in the Miami MSA, even though their area-weighted access to green space (i.e., local parks), is not significantly different from lower SES neighborhoods. Future research in places endowed with accessible coastal recreational amenities such as the Miami MSA should pursue integrated analyses of inequities in access to green and blue spaces.

In response to our second question—Are there neighborhood inequities in area-weighted park access within the Hispanic/Latino population based on country-of-origin in the Miami MSA?—we found that higher proportions of the tract population that were of Cuban and Venezuelan origin correlated with substantially reduced area-weighted park access (relative to the tract composition of White residents). Results for the other Hispanic/Latino ancestry subgroups had statistically non-significant associations with area-weighted park access, implying that Cuban and Venezuelan composition, in particular, drove the negative relationship with area-weighted park access we found for the Hispanic/Latino group. In a general sense, the intracategorical differences we found based on national origin within the Hispanic/Latino category were consistent with our initial hypothesis based on the findings of Chakraborty et al. [17], Grineski et al. [16], and Collins et al. [12], which focused on air pollution inequities.

This study extended the intracategorical EJ analysis approach to park equity research by investigating the effect that heterogeneous Hispanic/Latino ancestry has on park access. Our findings suggest that country of origin—which reflects differentiated immigration histories—intersects with Hispanic/Latino status to shape park access inequities in the Miami MSA. Based on our findings, future research using an intracategorical EJ approach is needed to study issues of park equity within the heterogenous Hispanic/Latino populations of large and diverse U.S. cities including (but not limited to) New York City, Los Angeles, and Chicago. U.S.-based park equity studies should also apply an intracategorical approach to examine inequities based on country-of-origin within the diverse Asian American population. Additionally, an intracategorical EJ approach should be applied to studies of park equity in world cities with highly ethnically diverse populations (e.g., London, Paris, Madrid, Hong Kong, Sydney). Inequities based on nativity (domestic vs. foreign birth), citizenship, language proficiency, SES, and age within highly heterogenous ethnicity categories should also be examined in future studies.

In terms of explanations for our intracategorical analysis results, we knew at the outset that the Hispanic/Latino population of the Miami MSA exhibited a differentiated residential geography (Figure 4) and stratified SES based on national origin; thus, we expected to uncover intracategorical park space inequities [16,60]. However, our findings that the most privileged national origin (Cuban) was associated with reduced neighborhood access to park space, while the most marginalized national origin (Mexican) was not, elude simple explanations. The two groups have varied settlement histories in the Miami MSA. Cuban Americans established themselves much earlier in neighborhoods within the core of Miami proper, which is endowed with relatively small parks, while Mexican migrants were more recently attracted to farm work along the metropolitan fringe, not far from larger acreage parks. Thus, we infer that Hispanic/Latino ethnicity has articulated in complex ways with national origin in the Miami MSA through time to produce neighborhood inequities in access to park space. While Cubans may represent a relatively privileged group based on national origin within the Hispanic/Latino population, they are nevertheless a minority immigrant group in the U.S. context. The environmental inequities that we and others have documented based on Cuban neighborhood composition in the Miami MSA may be predicated on their relatively longstanding experiences of ethnic marginalization and enclave formation, which occurred despite the political and economic privileges they have enjoyed based on national origin relative to other Hispanic/Latino people. In sum, these findings are likely attributable to the local historical geography of immigrant settlement and the tendencies toward neighborhood congregation and segregation based on the intersection of national origin and ethnic marginalization within the Miami MSA. Future longitudinal or historical research, such as that of Boone et al. [20], is needed to fully explain these intracategorical patterns of inequity.

Future studies can address the limitations of our methodology. Issues associated with our reliance on aggregated data—e.g., the modifiable areal unit problem (MAUP) and the problem of ecological inference—can only be fully addressed through analyses of individual or household-level data. As a measure of accessible park quality, our area-weighted park access measure can be extended in future research through the integration of other dimensions of park quality, such as tree canopy cover, lighting, facilities, signage, cleanliness, landscape design, and organized recreational activities [23]. Analysts can also extend our area-weighted park access measure in future research by integrating measures of pedestrian network distance and dasymetric population mapping techniques to estimate the residential locations of small area populations and their walking distances from parks more accurately [4,61].

## 5. Conclusions

We must take proactive steps to ameliorate park access inequities based on Black and Hispanic/Latino status in the Miami MSA and other contexts characterized by race-based park access inequities. Solutions include increasing public awareness about park (in)equity, organizing volunteer clean-up initiatives to improve the quality of rundown parks or making brownfields usable, and developing mini-parks, including green roofs [62]. The expansion of parks is a capital investment that is burdensome for municipal and county parks departments; as such, use fees, committed property taxes, and sales taxes typically fund parks. This revenue is often lower in racial/ethnic minority neighborhoods, partly due to historical discrimination and ongoing socioeconomic deprivation. However, new efforts can and should incorporate an equity focus into allocating and distributing local funds in the Miami MSA [2]. In such a social and fiscal context, park equity is achievable only if prioritized.

## Figures and Tables

**Table 1:** Descriptive statistics for analysis variables, Miami MSA census tracts (*n* = 1192).

List of Variables	Min	Max	Mean	SD
Area-Weighted Park Access (est. m^2^) (Dependent)	0.00	16,403,949	507,244	923,894
Proportion Hispanic	0.00	0.994	0.422	0.295
Proportion Mexican	0.00	0.409	0.023	0.040
Proportion Puerto Rican	0.00	0.154	0.036	0.028
Proportion Cuban	0.00	0.891	0.173	0.220
Proportion Colombian	0.00	0.285	0.039	0.040
Proportion Venezuelan	0.00	0.608	0.023	0.047
Proportion Other Hispanic	0.00	0.648	0.123	0.097
Proportion Non-Hispanic (NH) White	0.00	0.995	0.350	0.284
Proportion NH Black	0.00	0.964	0.188	0.241
Proportion NH Native American/Alaskan	0.00	0.059	0.001	0.004
Proportion NH Asian	0.00	0.147	0.022	0.024
Proportion NH Pacific Islander	0.00	0.081	0.000	0.003
Proportion NH Other/Multi Race	0.00	0.124	0.017	0.018
Proportion Under 18 Years of Age	0.00	0.435	0.192	0.073
Proportion Over 65 Years of Age	0.01	0.865	0.200	0.142
Proportion Renter-Occupied Housing	0.01	0.989	0.400	0.230
Median Household Income (2018, USD)	11,927	219,205	61,344	30,368

**Table 2:** Results of generalized estimating equations predicting area-weighted park access, Miami MSA census tracts (*n* = 1192).

	Model 1 ^1^			Model 2 ^2^		
Parameter	B (95% CI)	St. Error	*p*	B (95% CI)	St. Error	*p*
Intercept	13.442 (12.488, 14.396)	0.487	<0.001	13.260 (12.224, 14.297)	0.529	<0.001
Prop. Hispanic	−1.175 (−1.603, −0.746)	0.219	<0.001			
Prop. Mexican				0.590 (−1.186, 2.366)	0.906	0.515
Prop. Puerto Rican				−0.518 (−3.993, 2.956)	1.773	0.770
Prop. Cuban				−1.163 (−1.860, −0.467)	0.355	0.001
Prop. Colombian				0.408 (−1.723, 2.539)	1.087	0.707
Prop. Venezuelan				−3.207 (−5.216, −1.198)	1.025	0.002
Prop. Other Hispanic				−0.921 (−2.603, 0.761)	0.858	0.283
Prop. Black	−1.225 (−1.920, −0.530)	0.355	<0.001	−1.069 (−1.837, −0.302)	0.392	0.006
Prop. American Indian	−25.324 (−36.638, −12.011)	6.793	<0.001	−25.973 (−41.179, −10.749)	7.767	<0.001
Prop. Asian	−0.623 (−7.201, 5.954)	3.356	0.853	−0.690 (−7.441, 6.062)	3.445	0.841
Prop. Pacific Islander	−5.485 (−18.122, 7.152)	6.448	0.395	−7.028 (−23.681, 9.626)	8.497	0.408
Prop. Other/Multi-Race	7.494 (4.406, 10.582)	1.576	<0.001	7.307 (4.484, 10.129)	1.440	<0.001
Prop. Under 18 Years	1.595 (0.085, 3.106)	0.771	0.038	1.271 (−0.119, 2.662)	0.709	0.073
Prop. 65 Years and Older	−0.136 (−1.471, 1.145)	0.667	0.807	−0.063 (−1.392, 1.266)	0.678	0.926
Prop. Renter-Occupied	0.205 (−0.329, 0.739)	0.272	0.452	0.242 (−0.326, 0.809)	0.289	0.404
Median Household Income	<0.000 (<0.000, >0.000)	>0.001	0.617	<0.000 (<0.000, >0.000)	>0.000	0.964

^1^ Model 1 uses an exchangeable correlation structure, a gamma distribution, and a log link function; quasi-likelihood under independence criterion (QIC) goodness-of-fit = 2,649,099. ^2^ Model 2 uses an exchangeable correlation structure, a Tweedie (index parameter = 1.5) distribution, and log link function; QIC = 1,070,735,562. Notes: B = beta coefficient, CI = confidence interval, St. = standard, Prop. = proportion.
